# Performance evaluation of serum PIVKA‐II measurement using HISCL‐5000 and a method comparison of HISCL‐5000, LUMIPULSE G1200, and ARCHITECT i2000

**DOI:** 10.1002/jcla.22921

**Published:** 2019-05-26

**Authors:** Mi Ra Ryu, Eun‐Suk Kang, Hyung‐Doo Park

**Affiliations:** ^1^ Department of Laboratory Medicine and Genetics Samsung Medical Center, Sungkyunkwan University School of Medicine Seoul Korea

**Keywords:** analytical performance, ARCHITECT i2000, HISCL‐5000, LUMIPULSE G1200, PIVKA‐II

## Abstract

**Background:**

Protein induced by vitamin K antagonist‐II (PIVKA‐II), in addition to alpha‐fetoprotein, is a useful tumor marker for diagnosis of hepatocellular carcinoma (HCC). We evaluated the analytical performance of the HISCL‐5000 analyzer (Sysmex Corporation) in the measurement of serum PIVKA‐II.

**Methods:**

We evaluated the precision and linearity of PIVKA‐II assays using the HISCL‐5000 analyzer. Methods using HISCL‐5000, LUMIPULSE G1200 (Fujirebio Diagnostics), and ARCHITECT i2000 (Abbott Diagnostics) were compared according to the guidelines of the Clinical and Laboratory Standards Institute. A total of 501 subjects (median age 59 years, age range 24‐90 years) were enrolled. Among them, 335 were HCC patients, 46 were patients with non‐HCC liver disease, and 120 were healthy individuals. Non‐HCC liver disease included liver cirrhosis, chronic hepatitis, HBV or HCV carrier, hepatic adenoma, and intrahepatic cholangiocarcinoma.

**Results:**

Repeatability (%CV) in low‐ and high‐level controls and pooled serum was 2.81%‐10.30%, and within‐laboratory precision was 4.24%‐8.86%. In a linearity test, the coefficient of determination (*R* ^2^) was 0.9957, ranging from 11 to 69 897 mAU/mL. In comparison, the coefficient of correlation (*r*) was 0.9561‐0.9644, agreement was 93.4%‐97.6%, and the *κ* value was 0.855‐0.945 among the three analyzers. About 99.2% of healthy individuals and 84.8% of non‐HCC liver disease patients were below the cutoff value (40 mAU/mL) on HISCL‐5000.

**Conclusions:**

A PIVKA‐II assay using HISCL‐5000 showed acceptable analytical performance including precision, linearity, and method comparison. This indicates that HISCL‐5000 can be potentially helpful in clinical laboratories.

## 1 INTRODUCTION

Hepatocellular carcinoma (HCC) is the fourth most common cancer in men and the sixth in women; in Korea, it is the second most common cause of death from cancer in men and the fourth in women.^1^ HCC has a mortality rate higher than that of other cancers in patients in their 40s and 50s. It is important to detect early‐stage HCC in high‐risk groups such as those with HBV infection, HCV infection, or cirrhosis. Several serum markers for diagnosing liver fibrosis or cirrhosis are known, and the diagnosis of HCC is based on pathology or imaging studies.^2^, ^3^ Regular surveillance is warranted in these high‐risk groups.^2^, ^4^, ^5^ HCC surveillance programs for high‐risk groups have been performed using ultrasound and serum alpha‐fetoprotein (AFP) level in Korea.^1^ However, serum AFP level is normal in some small HCC and can be nonspecifically elevated in patients with non‐HCC liver disease.^2^ A recent Japanese guideline indicates the combined use of AFP and protein induced by vitamin K antagonist‐II (PIVKA‐II) for diagnosis of HCC.^6^ Several studies also have reported the clinical usefulness of PIVKA‐II.^7^, ^8^, ^9^

PIVKA‐II, which is also referred to as des‐gamma‐carboxy prothrombin, is widely used as a tumor marker in addition to AFP for diagnosis of HCC. PIVKA‐II is an abnormal prothrombin produced in the absence of vitamin K or when its activity is decreased in liver cells. Normally, 10 glutamic acids (Glu) in the N‐terminal domain are converted to γ‐carboxyglutamic acids (Gla) by a vitamin K–dependent carboxylase. In the presence of vitamin K deficiency, all or some of the 10 Glu cannot be converted to Gla, and abnormal prothrombin can be secreted into the blood.^10^, ^11^

In 1984, Liebman et al^12^ first reported serum PIVKA‐II level to be significantly elevated in HCC patients. Many studies have reported that PIVKA‐II is a good and effective biomarker for detection and surveillance of HCC. The incidence of HCC is highest in East Asia, sub‐Saharan Africa, and Melanesia, where around 83% of cases occur.^13^ PIVKA‐II tests have routinely been used to screen for HCC in addition to ultrasound in Japan, whereas they are not recommended in Europe and America.^14^, ^15^ PIVKA‐II has been considered an essential marker for HCC surveillance in Asia.^16^

The present study aimed to evaluate the analytical performance of serum PIVKA‐II measurement using an HISCL‐5000 analyzer (Sysmex Corporation) and to compare concentrations of PIVKA‐II measured using HISCL‐5000, LUMIPULSE G1200, and ARCHITECT i2000.

## 2 MATERIALS AND METHODS

### 2.1 Study population

We collected serum from 501 individuals between October 2017 and December 2017 at Samsung Medical Center. Among a total of 501 subjects, 335 had HCC, 46 had non‐HCC liver disease, and 120 were healthy individuals who attended a regular health checkup (Table 1). Non‐HCC liver disease included liver cirrhosis, chronic hepatitis, HBV or HCV carrier, hepatic adenoma, and intrahepatic cholangiocarcinoma. Of the 335 samples from HCC patients, five were collected before therapeutic intervention, and 328 were from treated patients. The diagnosis of HCC is based on pathology or imaging studies in high‐risk groups. A total of 355 HCC patients in this study were high‐risk groups such as those with HBV infection, HCV infection, and cirrhosis, and all of them were diagnosed with HCC by image study. Pathologic examination was performed in some of these patients with surgical treatment after diagnosis. This study was approved by the Ethics Committee at Samsung Medical Center.

**Table 1:** Characteristics of patients enrolled in the study (n = 501)

Variable	HCC (n = 335)	Non‐HCC liver disease^a^ (n = 46)	Healthy control (n = 120)
Age (y), median (range)	62 (33‐90)	60 (35‐86)	49 (24‐83)
Sex, n (%)			
Male	279 (83.3)	33 (71.7)	36 (30.0)
Etiology, n (%)			
HBV	279 (83.3)	33 (71.7)	NA
HCV	22 (6.6)	3 (6.5)	NA
HBV + HCV	1 (0.3)	0 (0.0)	NA
Alcohol	13 (3.9)	7 (15.2)	NA
Other	20 (6.0)	3 (6.5)	NA

Abbreviations: HBV, hepatitis B virus; HCC, hepatocellular carcinoma; HCV, hepatitis C virus; NA, not applicable.

a Non‐HCC liver disease includes liver cirrhosis, chronic hepatitis, HBV or HCV carrier, hepatic adenoma, and intrahepatic cholangiocarcinoma.

### 2.2 Instruments

We evaluated the basic performance of PIVKA‐II assays using the HISCL‐5000 analyzer, a fully automated immunochemistry analyzer that employs a chemiluminescence enzyme immunoassay (CLEIA) methodology with a two‐step sandwich immunoassay. The primary antibody was anti‐PIVKA‐II mouse monoclonal antibody (MU‐3 antibody), and the secondary antibody was anti‐prothrombin mouse monoclonal antibody. As a control method, a CLEIA on LUMIPULSE G1200 (Fujirebio Diagnostics) and a chemiluminescent microparticle immunoassay (CMIA) on ARCHITECT i2000 (Abbott Diagnostics) were performed according to the manufacturers' instructions. The primary antibody of the control reagents was anti‐PIVKA‐II mouse monoclonal antibody (MU‐3 antibody) for LUMIPULSE G1200 and anti‐PIVKA‐II mouse monoclonal antibody (3C10 antibody) for ARCHITECT i2000. The secondary antibody of the control reagents was anti‐prothrombin rabbit polyclonal antibody for LUMIPULSE G1200 and anti‐prothrombin mouse monoclonal antibody for ARCHITECT i2000. The analytical measurement ranges of HISCL‐5000, LUMIPULSE G1200, and ARCHITECT i2000 were 5‐75 000 mAU/mL, 5‐75 000 mAU/mL, and 20‐30 000 mAU/mL, respectively. The cutoff value for PIVKA‐II was 40 mAU/mL in all three analyzers.

### 2.3 Precision

Precision was assessed using two levels of quality control material and pooled serum, according to the Clinical and Laboratory Standards Institute's (CLSI) EP05‐A3 guideline.^17^ High‐ and low‐level quality control substances for PIVKA‐II were provided by the manufacturer. Repeatability and within‐laboratory precision (%CV) were assessed by measuring twice each day for 20 days, and each test value was determined as the mean value of the two measurements.

### 2.4 Linearity

Linearity was evaluated according to the CLSI EP06‐A guideline.^18^ The high‐ and low‐concentration control materials that were close to the upper and lower limits of the measurement range, respectively, were used. Linearity was evaluated at mixture ratios of 4:0, 3:1, 2:2, 1:3, and 0:4. The test was repeated four times for each of the five concentrations, and linearity was evaluated as the coefficient of determination (*R* ^2^).

### 2.5 Comparison

The comparison test was performed to compare the HISCL‐5000 assay to the LUMIPULSE G1200 assay and ARCHITECT i2000 assay, according to the CLSI EP09‐A3 guideline.^19^ A total of 501 serum samples were aliquoted into three fractions, which were separately tested using each of the three instruments. The comparison was performed using the coefficient of correlation (*r*), the percentages of difference and agreement, and *κ* values between the results of the respective analyzers.

## 3 RESULTS

**Figure 1:**
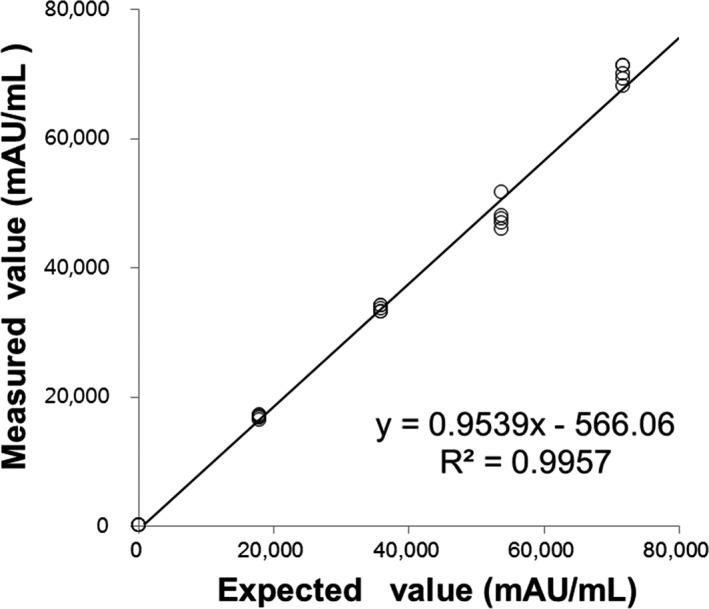
Linearity curve of measured value vs expected value for measurement of serum PIVKA‐II concentration by HISCL‐5000

Repeatability (%CV) in low‐ and high‐level controls and pooled serum was 2.81%, 3.17%, and 10.30%, respectively, and within‐laboratory precision was 4.33%, 4.24%, and 8.86%, respectively. In the linearity test, *R* ^2^ was 0.9957, ranging from 11 to 69 897 mAU/mL (Figure 1).

**Figure 2:**
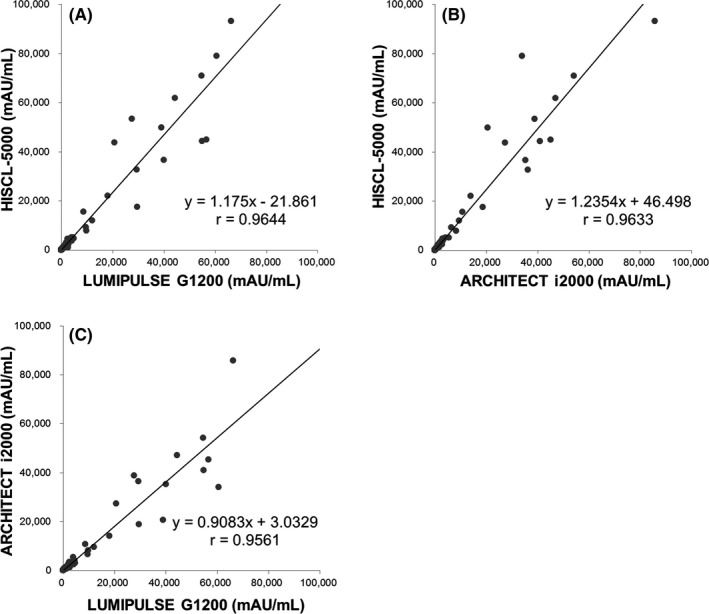
Comparison of three analyzers for measurement of serum PIVKA‐II concentration. (A) LUMIPULSE G1200 vs HISCL‐5000, (B) ARCHITECT i2000 vs HISCL‐5000, and (C) LUMIPULSE G1200 vs ARCHITECT i2000

**Figure 3:**
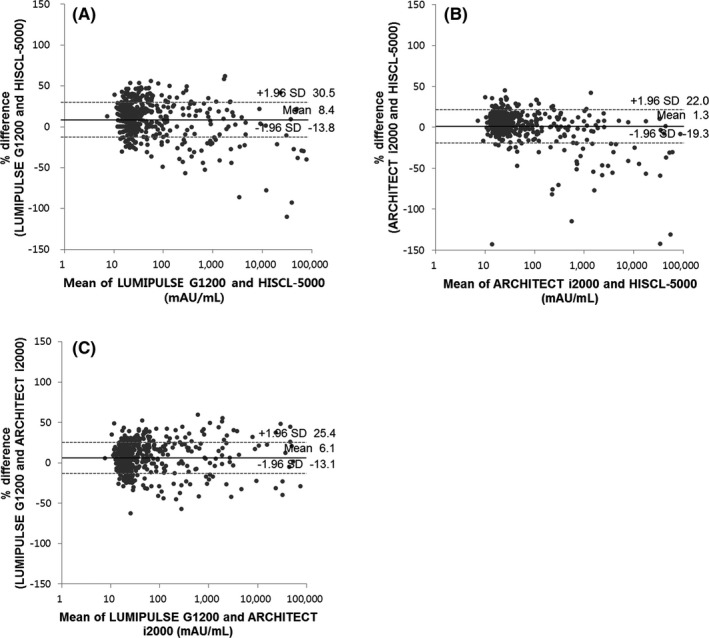
Bland‐Altman plots of the paired difference in concentration of serum PIVKA‐II measured using the three analyzers. (A) LUMIPULSE G1200 vs HISCL‐5000, (B) ARCHITECT i2000 vs HISCL‐5000, and (C) LUMIPULSE G1200 vs ARCHITECT i2000

Figure 2 shows a coefficient of comparison (*r*) among the three analyzers of 0.9561‐0.9644. Table 2 shows a relatively high percentage of specimens negative in HISCL‐5000 and positive in LUMIPULSE G1200 (6.4%, 32/501). Table 3 shows that the agreements were 93.4%, 97.6%, and 94.6%, and the *κ* values were 0.855, 0.945, and 0.882 between LUMIPULSE G1200 and HISCL‐5000, between ARCHITECT i2000 and HISCL‐5000, and between LUMIPULSE G1200 and ARCHITECT i2000, respectively. In the Bland‐Altman plots of the paired differences (Figure 3), the mean difference was 1.3%‐8.4% among the three different analyzers. In LUMIPULSE G1200 vs HISCL‐5000 and ARCHITECT i2000 vs HISCL‐5000, there were larger differences at higher concentrations, while the differences in LUMIPULSE G1200 vs ARCHITECT i2000 were not significant.

**Table 2:** Qualitative comparisons between LUMIPULSE G1200, ARCHITECT i2000, and HISCL‐5000

	HISCL‐5000 (cutoff: 40 mAU/mL)		
	Positive	Negative	Total
LUMIPULSE G1200 (cutoff: 40 mAU/mL)			
Positive	157	32	189 (37.7%)
Negative	1	311	312 (62.3%)
Total	158 (31.5%)	343 (68.5%)	501
ARCHITECT i2000 (cutoff: 40 mAU/mL)			
Positive	154	8	162 (32.3%)
Negative	4	335	339 (67.7%)
Total	158 (31.5%)	343 (68.5%)	501

**Table 3:** Qualitative agreement between LUMIPULSE G1200, ARCHITECT i2000, and HISCL‐5000

	Agreement (%)	95% confidence interval	*κ* value
All samples (n = 501)			
LUMIPULSE G1200 vs HISCL‐5000	93.4	0.808‐0.902	0.855
ARCHITECT i2000 vs HISCL‐5000	97.6	0.914‐0.976	0.945
LUMIPULSE G1200 vs ARCHITECT i2000	94.6	0.839‐0.925	0.882
HCC (n = 335)			
LUMIPULSE G1200 vs HISCL‐5000	91.6	0.775‐0.892	0.834
ARCHITECT i2000 vs HISCL‐5000	96.7	0.895‐0.972	0.934
LUMIPULSE G1200 vs ARCHITECT i2000	93.1	0.810‐0.917	0.863
Non‐HCC liver disease (n = 46)			
LUMIPULSE G1200 vs HISCL‐5000	87.0	0.555‐1.000	0.785
ARCHITECT i2000 vs HISCL‐5000	97.8	0.767‐1.000	0.920
LUMIPULSE G1200 vs ARCHITECT i2000	95.7	0.677‐1.000	0.862
Healthy control (n = 120)			
LUMIPULSE G1200 vs HISCL‐5000	98.3	−0.106‐1.000	0.494
ARCHITECT i2000 vs HISCL‐5000	100.0	1.000‐1.000	1.000
LUMIPULSE G1200 vs ARCHITECT i2000	98.3	−0.106‐1.000	0.494

**Figure 4:**
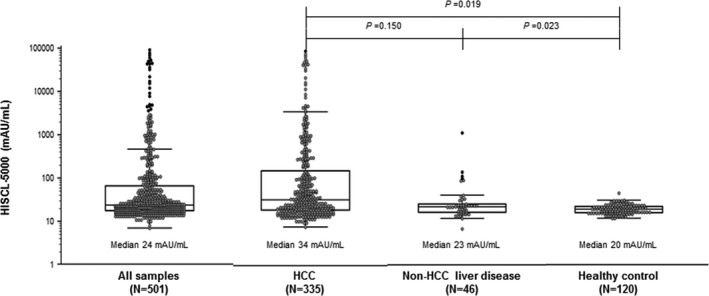
Level of serum PIVKA‐II in different patient groups. Gray points refer to serum values, and horizontal lines below and above refer to the 25th and 75th percentile values, respectively. Horizontal lines within boxes indicate median levels. *P* values were calculated by Mann‐Whitney tests between two columns

Figure 4 shows the distribution of serum PIVKA‐II level by HISCL‐5000 among the various subject groups, consisting of HCC, non‐HCC liver disease, and healthy individuals. Median serum level of PIVKA‐II in the HCC group and the non‐HCC liver disease group was significantly higher than that in healthy individuals (Mann‐Whitney, *P* = 0.019 and *P* = 0.023, respectively). The range of PIVKA‐II level using the HISCL‐5000 analyzer was 7‐93 121 mAU/mL in the HCC group, 7‐1158 mAU/mL in the non‐HCC liver disease group, and 12‐47 mAU/mL in healthy individuals. The results for the other two analyzers are summarized in Table S1. Of the 120 healthy individuals, 119 (99.2%) were below the cutoff value in HISCL‐5000, 117 (97.5%) in LUMIPULSE G1200, and 119 (99.2%) in ARCHITECT i2000.

## 4 DISCUSSION

Enzyme‐linked immunosorbent assay (ELISA) using an anti‐PIVKA‐II monoclonal antibody has been widely used in clinical laboratories.^16^, ^20^ Fully automated analyzers using various methodologies such as CLEIA or CMIA—for example, LUMIPULSE G1200 and ARCHITECT i2000—have been introduced and used clinically, and their analytical performances have been evaluated.^16^, ^21^ Recently, Sysmex Corporation has released a newly developed PIVKA‐II assay kit using the HISCL‐5000 analyzer.

This study showed that serum PIVKA‐II by HISCL‐5000 had acceptable precision and linearity and was comparable to LUMIPULSE G1200 and ARCHITECT i2000. Overall, we found a high degree of agreement among LUMIPULSE G1200, ARCHITECT i2000, and HISCL‐5000 (agreement: 93.4%‐97.6%; *κ* value: 0.855‐0.942). Among the three groups of subjects, the HCC group showed the lowest agreement among the analyzers (agreement: 91.6%‐93.1%; *κ* value: 0.833‐0.862) and had a wider range (8‐93 121 mAU/mL) than the other groups. One consideration for this could be that the HCC group included patients prior to treatment and immediately after therapeutic intervention and/or patients with recurrence of HCC, and the level of serum PIVKA‐II is known to have a close correlation with tumor size.^22^ For 36 patients, discordant results in any of the three analyzer pairs were mostly due to PIVKA‐II values around or near the cutoff value (40 mAU/mL), and most of them (31/36) were from the HCC group.

Larger differences were observed at higher concentrations between LUMIPULSE G1200 and HISCL‐5000 and between ARCHITECT i2000 and HISCL‐5000. This could be caused by use of different antibodies or interference of endogenous substances in the different patient samples. Different antibodies recognize different amino acid residues in the Gla domain,^23^ and conformational change of the antigens may be responsible for the differences.

As shown in Figure 4, there was no significant difference in median serum level of PIVKA‐II between the HCC group and the non‐HCC liver disease group (Mann‐Whitney, *P* = 0.150). Serum PIVKA‐II level may be elevated in not only HCC but also liver cirrhosis, because of alterations in vitamin K production secondary to cholestasis, malnutrition, or use of medication.^24^, ^25^ In this study, seven of the 46 non‐HCC liver disease patients were above the cutoff value (40 mAU/mL), and they were all liver cirrhosis patients. Although they showed higher values, serum PIVKA‐II level range at higher concentrations in the HCC group was much wider than that of the non‐HCC liver disease group (8‐125 035 mAU/mL and 7‐1158 mAU/mL, respectively). However, there was no significant difference in median serum level of PIVKA‐II between the two groups, because 328 of the 335 patients (97.9%) in the HCC group had a history of treatment, suggesting that their median serum PIVKA‐II levels were decreased. Nonetheless, it should be noted that healthy individuals have significantly lower serum PIVKA‐II level than the HCC group and the non‐HCC liver disease group.

Of the 120 healthy individuals, only one showed a false high value, having a PIVKA‐II level near the cutoff value in all three analyzers (HISCL‐5000, 47 mAU/mL; LUMIPULSE G1200, 46 mAU/mL; ARCHITECT i2000, 55 mAU/mL). False‐positive results of PIVKA‐II values are known to occur when the following factors are present: vitamin K deficiency, administration of warfarin, primary gastric adenocarcinoma, graft rejection after liver transplantation, acute hepatic failure, malnutrition, use of antibiotics that alter gut flora, underlying renal failure, coexisting inflammatory bowel disease, and alcoholic liver disease.^26^ The false higher PIVKA‐II level near the cutoff value in the patient could be due to a small margin of variability with the dichotomous cutoff of 40 mAU/mL.

As mentioned above, because the HCC group included patients of various disease statuses with unknown baseline PIVKA‐II level before therapeutic intervention, there were limitations in determining the diagnostic sensitivity and other clinical usefulness in this study. However, there have been many reports about the role of PIVKA‐II as an indicator of treatment efficacy in HCC patients. Park et al^27^ have reported that maximum PIVKA‐II level reductions from baseline were 85.3% and 80.6% after transarterial chemoembolization (TACE) in patients with complete response and partial response, respectively. In a study by Lee et al,^28^ patients with a baseline PIVKA‐II level >200 mAU/mL showed a significant correlation between overall survival and PIVKA‐II response defined as a reduction greater than 50% from baseline after TACE. Yamamoto et al^22^ have reported that positive PIVKA‐II values became negative at 6 months posthepatectomy in 99.6% of patients.

In conclusion, the PIVKA‐II assay using HISCL‐5000 showed acceptable analytical performance including precision, linearity, and method comparison, and there was a high degree of agreement among the three analyzers. This indicates that HISCL‐5000 can be potentially helpful in clinical laboratories.

## CONFLICT OF INTEREST

The authors declared no potential conflicts of interest with respect to the research, authorship, and/or publication of this article.

## AUTHOR CONTRIBUTIONS

EK and HP contributed equally to this work. HP conceived and designed the study. MR, EK, and HP performed the experiments and analyzed the data. MR wrote the manuscript. EK and HP reviewed and modified the manuscript.

## ETHICAL APPROVAL

This study was approved by the Ethics Committee of Samsung Medical Center (reference number: 2017‐07‐153‐001). Guarantor: Hyung‐Doo Park.
