# Supplementary material for: Performance evaluation of serum PIVKA‐II measurement using HISCL‐5000 and a method comparison of HISCL‐5000, LUMIPULSE G1200, and ARCHITECT i2000
Source: J Clin Lab Anal. 2019 May 26;33(6):e22921. doi: 10.1002/jcla.22921 (PMC6642327; doi:10.1002/jcla.22921)
Supplement: Supplementary file 1 [file JCLA-33-e22921-s001.docx]

Table S1. Summary statistics between LUMIPULSE G1200, ARCHITECT i2000 and HISCL-5000 in different patient groups (mAU/mL)

|  | HCC (n=335) | | |  | Non-HCC liver disease† (n=46) | | |  | Healthy control (n=120) | | |
| --- | --- | --- | --- | --- | --- | --- | --- | --- | --- | --- | --- |
|  | HISCL-5000 | LUMIPULSE G1200 | ARCHITECT i2000 |  | HISCL-5000 | LUMIPULSE G1200 | ARCHITECT i2000 |  | HISCL-5000 | LUMIPULSE G1200 | ARCHITECT i2000 |
| Lowest value | 8 | 13 | 9 |  | 7 | 8 | 8 |  | 12 | 12 | 8 |
| Highest value | 125,035 | 66,254 | 106,642 |  | 1,158 | 1,020 | 1,176 |  | 47 | 46 | 55 |
| Mean | 2,719 | 2,027 | 2,157 |  | 56 | 59 | 61 |  | 21 | 21 | 22 |
| Median | 34 | 43 | 36 |  | 23 | 25 | 24 |  | 20 | 20 | 21 |

Abbreviation: HCC, hepatocellular carcinoma

† Non-HCC liver disease include liver cirrhosis, chronic hepatitis, HBV or HCV carrier, hepatic adenoma and intrahepatic cholangiocarcinoma.
